# Research on Robot Obstacle Avoidance and Generalization Methods Based on Fusion Policy Transfer Learning

**DOI:** 10.3390/biomimetics10080493

**Published:** 2025-07-25

**Authors:** Suyu Wang, Zhenlei Xu, Peihong Qiao, Quan Yue, Ya Ke, Feng Gao

**Affiliations:** 1 School of Mechanical and Electrical Engineering, China University of Mining and Technology, Beijing 100083, China; wsy@cumtb.edu.cn (S.W.); xzl@student.cumtb.edu.cn (Z.X.); zqt2300402058@student.cumtb.edu.cn (P.Q.); yq@student.cumtb.edu.cn (Q.Y.); zqt2400402060@student.cumtb.edu.cn (Y.K.); 2 Institute of Intelligent Mining and Robotics, China University of Mining and Technology, Beijing 100083, China; 3 Beijing Huatie Information Technology Co., Ltd., Beijing 100081, China; 4 Signal & Communication Research Institute, China Academy of Railway Sciences Corporation Limited, Beijing 100081, China

**Keywords:** bio-inspired, policy fusion networks, bio-inspired radar perception features, ineffective behavior recognition, transfer learning

## Abstract

In nature, organisms often rely on the integration of local sensory information and prior experience to flexibly adapt to complex and dynamic environments, enabling efficient path selection. This bio-inspired mechanism of perception and behavioral adjustment provides important insights for path planning in mobile robots operating under uncertainty. In recent years, the introduction of deep reinforcement learning (DRL) has empowered mobile robots to autonomously learn navigation strategies through interaction with the environment, allowing them to identify obstacle distributions and perform path planning even in unknown scenarios. To further enhance the adaptability and path planning performance of robots in complex environments, this paper develops a deep reinforcement learning framework based on the Soft Actor–Critic (SAC) algorithm. First, to address the limited adaptability of existing transfer learning methods, we propose an action-level fusion mechanism that dynamically integrates prior and current policies during inference, enabling more flexible knowledge transfer. Second, a bio-inspired radar perception optimization method is introduced, which mimics the biological mechanism of focusing on key regions while ignoring redundant information, thereby enhancing the expressiveness of sensory inputs. Finally, a reward function based on ineffective behavior recognition is designed to reduce unnecessary exploration during training. The proposed method is validated in both the Gazebo simulation environment and real-world scenarios. Experimental results demonstrate that the approach achieves faster convergence and superior obstacle avoidance performance in path planning tasks, exhibiting strong transferability and generalization across various obstacle configurations.

## 1. Introduction

With the continued advancement of research in biomimetics, an increasing number of engineering systems have begun to draw inspiration from the perception, decision-making, and locomotion mechanisms of biological organisms in nature, aiming to enhance adaptability and intelligence in complex environments. For instance, simulated lemming migration behaviors have been applied to unmanned aerial vehicle obstacle avoidance, enabling flexible navigation with limited sensory input [1]; the migratory characteristics of antelopes have been used to guide obstacle avoidance and path planning in medical patient transport AGVs [2]; and humans are particularly adept at integrating long-term experience with real-time perception to dynamically adjust their actions. These natural phenomena reveal a key insight: organisms operating under incomplete perception and uncertain information can often achieve efficient, goal-directed behavior through the integration of experience and perception. This biomimetic principle provides important theoretical support for intelligent path planning in ground mobile robots.

Ground mobile robots have been widely applied in various real-world scenarios such as warehouse logistics [3], intelligent inspection [4], rescue operations [5], and service applications [6]. With the growing demand for intelligent systems and the increasing complexity of operating environments, breakthroughs and innovations in the core technologies of mobile robots have become increasingly critical. Among them, path planning is a key component for achieving autonomous navigation, with the primary goal of generating an optimal path from a starting point to a target while satisfying specific constraints [7]. In practical applications, robots often operate in unknown or dynamic environments where complete prior map information cannot be reliably assumed [8]. Ground robots equipped with advanced path planning capabilities can not only significantly reduce task execution time but can also effectively enhance operational safety and stability. In real-world applications, robots often operate in unknown or dynamic environments, making it difficult to rely on complete prior map information. Therefore, enabling robots to autonomously avoid obstacles and navigate toward targets in complex environments has become a core challenge and a key direction for future research in ground mobile robotics.

Path planning algorithms can be broadly categorized into two main types: classical methods and learning-based approaches. Classical algorithms, such as graph search, sampling-based methods, and bio-inspired intelligent algorithms, are known for their clear modeling logic and interpretability. Graph search algorithms, including Dijkstra’s algorithm [9], A algorithm [10], Jump Point Search (JPS) [11], and D algorithm [12], are commonly applied in static path planning scenarios. Bio-inspired intelligent algorithms imitate the adaptive behaviors of animals and plants in nature and include methods such as Genetic Algorithm (GA) [13], Ant Colony Optimization (ACO) [14], and Particle Swarm Optimization (PSO) [15]. Owing to their biomimetic nature, these algorithms typically offer fast convergence; however, they are prone to becoming trapped in local optima.

Learning-based approaches, represented by deep learning and reinforcement learning, have developed rapidly in recent years. A key advantage of these methods lies in their ability to autonomously learn policies through interaction with the environment, enabling efficient path planning even in unknown environments without relying on precise mapping [16]. Common methods include Deep Q-Networks (DQN) [17], Deep Deterministic Policy Gradient (DDPG) [18], Twin Delayed DDPG (TD3) [19], and Soft Actor–Critic (SAC) [20]. The progressive evolution from DQN and DDPG to TD3 has significantly improved policy stability and training efficiency by mitigating the overestimation problem in value functions. SAC further enhances policy expressiveness by incorporating the maximum entropy reinforcement learning framework and has demonstrated excellent performance across various continuous control tasks. Compared with traditional path planning techniques, deep reinforcement learning (DRL) methods show notable advantages in handling complex and dynamic environments. However, despite the remarkable progress achieved by these algorithms, they often require large amounts of interaction data for training, suffer from slow convergence, and are highly sensitive to environmental changes, making it difficult to directly transfer them to new scenarios [21].

This lack of generalization capability limits the widespread application of reinforcement learning algorithms in complex environments. To enhance policy adaptability and training efficiency in dynamic scenarios, transfer learning has been introduced into reinforcement learning frameworks as an effective means of knowledge transfer. By leveraging policy experience acquired in a source domain and transferring it to a related target domain, agents can significantly reduce exploration costs in the target environment, thereby accelerating policy convergence [22]. However, several key challenges remain. The most prominent among them is the negative transfer problem: when there is a substantial discrepancy between the source and target domains, incorrect or irrelevant knowledge from the source policy may be mistakenly transferred, disrupting policy optimization and even degrading overall performance [23]. In addition, distributional differences between the source and target domains—in terms of sensory features, action spaces, or environmental dynamics—can also significantly hinder the effectiveness of transfer learning [24].

To address the challenges of policy transfer difficulties, heavy perception computation, and sparse reward feedback in reinforcement learning-based path planning, there is an urgent need to develop an efficient deep reinforcement learning algorithm. Accordingly, this paper proposes a deep reinforcement learning approach based on the SAC, aiming to improve policy transfer stability, perception efficiency, and reward feedback rationality in complex environments. The specific contributions are as follows:

1. We propose a transfer learning method based on a policy fusion network. By integrating a pretrained policy (representing prior experience) with a current policy (reflecting real-time perception), this approach enables the robot to adaptively learn new behaviors in novel environments;
2. We designed a bio-inspired LiDAR perception optimization method, which performs sparse sampling and regional partitioning on high-dimensional LiDAR data to extract key environmental features. By computing statistical indicators only within critical regions, the method improves perception efficiency and reduces the computational burden on the policy networks;
3. A reward function based on an ineffective behavior identification mechanism is constructed to dynamically evaluate whether the robot is approaching the target point, thereby reducing ineffective exploration and preventing the robot from falling into local optima.

## 2. Related Work

In reinforcement learning-based path planning tasks, policies often need to be trained from scratch in new environments, which results in long training durations, high sample requirements, and increased vulnerability to interference from multiple obstacles during the convergence process. As an effective approach to improving policy generalization and learning efficiency, transfer learning enables agents to reduce training costs in the target domain by transferring knowledge learned from source environments, thereby enhancing performance in new scenarios. In 2023, Du et al. [25] quantitatively measured the similarity between various source and target environments based on UAV flight trajectories and selected the most similar source scenario for transfer, effectively reducing the potential negative effects of transfer learning. In 2024, they further proposed a transfer learning algorithm tailored for graph neural networks (GNNs) [26]. By freezing the parameters of aggregation layers and fine-tuning the rest of the model, the method preserved the general feature extraction capability for graph structures while reducing the number of trainable parameters. Li et al. [27] used pretrained model parameters as the initial weights in the target environment and maintained a certain level of exploration capability to accommodate variations, enabling knowledge re-adaptation and policy fine-tuning, and thereby improving the transferability across different tasks. Lan et al. [28] introduced a novel transfer reinforcement learning framework with an adaptive parameter layer to dynamically regulate the adaptation ratio during transfer. Their method first built a pretrained model and then used an enhanced low-rank adaptation algorithm to effectively integrate prior knowledge into the main training process. Most existing approaches rely on measuring the similarity between source and target environments or designing suitable parameter transfer strategies. However, they often fail to capture the key decision-making mechanisms during policy transfer, which may lead to transfer failure or even negative transfer. Additionally, some methods attempt to improve adaptability by increasing model complexity or enhancing exploration capabilities, but this results in additional computational costs and model overhead.

As one of the core perception inputs in the state space, LiDAR information plays a crucial role in obstacle detection. Processing LiDAR data has thus become an essential approach for robots to obtain more accurate environmental information. Wang et al. [29] transformed LiDAR readings into distance measurements from obstacles to specific directions around the robot, thereby eliminating the influence of the robot’s physical shape on the decision-making networks and enabling more precise obstacle avoidance and improved maneuverability under extreme conditions. Zhang et al. [30] proposed an input preprocessing method named IPAPRec, which employs an adaptive reciprocal function to preprocess LiDAR readings, emphasizing important short-range values in laser scans and compressing less relevant long-range values, thus addressing limitations in conventional LiDAR representations. Jorge de Heuvel et al. [31] introduced a spatiotemporal attention module based on 2D LiDAR sensor data to infer the relative importance of different observation areas in terms of proximity to the target and obstacle movement trends, thereby improving overall navigation performance in dynamic scenarios. In addition to processing LiDAR data alone, several multimodal approaches have also been proposed. Ou Yang et al. [32] fused data from LiDAR sensors and cameras and combined odometry readings with target coordinates to construct an instantaneous state of the decision-making environment, achieving complementary sensing through multi-sensor fusion. Tan et al. [33] designed a multimodal perception module based on image and LiDAR data, employing semantic segmentation techniques to bridge the gap between simulated and real-world environments and enhance the robot’s perception capability. However, although these existing methods have achieved some progress in improving perception accuracy and multimodal fusion, most of them still rely on direct LiDAR data processing or deep networks for feature extraction, without fully considering the task relevance and regional priority of perception information. With high-dimensional sensory input, the neural networks requires larger capacity and more frequent updates, leading to increased computational cost and slower policy convergence, especially in terms of the number of episodes needed to reach stable performance.

Meanwhile, the design of the reward function plays a critical role in determining both the convergence speed and the final performance of policy learning. Numerous studies have been conducted to improve reward mechanisms. Yuan et al. [34] proposed a dynamic composite reward function that integrates heuristic distance, azimuth, and turning penalties, providing the robot with rich and informative reward signals. Peng et al. [35] introduced a dense stage-based reward scheme composed of posture, stride, and phase-specific soft and hard incentives, which collectively reduce blind exploration. Yan et al. [36] enhanced the reward function by incorporating directional reward components, linear velocity factors, and safety performance factors as coefficients. They used heading, speed, and safety as evaluation metrics and adopted information entropy to dynamically adjust the influence weights of multi-objective components in the reward function to improve robotic performance. Sheng et al. [37] proposed a dynamically adaptive reward function based on non-sparse design, in which the relative weights of distance, step, and collision rewards vary across training stages, providing gradually evolving guidance to improve overall performance. In recent years, significant efforts have been made to refine and reconstruct reward functions, thereby improving the efficiency of policy learning. However, in complex environments or scenarios with dense obstacles, existing reward mechanisms still face the issue of local optima. Moreover, overly dense reward structures may lead to a shift in the learning focus of the policy, causing the agent to concentrate on secondary objectives rather than the primary task objective, which disperses learning attention and ultimately reduces the overall effectiveness of policy training.

To address the issues of low transfer efficiency, limited perception capability, and dispersed learning focus in reinforcement learning-based robotic path planning, this paper proposes a method built upon the original SAC framework. The approach introduces fusion policy networks, optimizes the structure of LiDAR observation features, and incorporates an ineffective behavior identification mechanism to refine the reward function. Existing transfer learning methods often reuse pretrained networks or fine-tune policies, but they lack explicit mechanisms to integrate prior and current policies during inference. The method proposed in this paper achieves action-level fusion, enabling the dynamic integration of prior knowledge and current policy in a flexible and adaptive manner, rather than merely performing parameter- or feature-level transfer. These improvements collectively enhance policy learning efficiency and transfer performance in complex environments.

## 3. Methods

**Figure 1:**
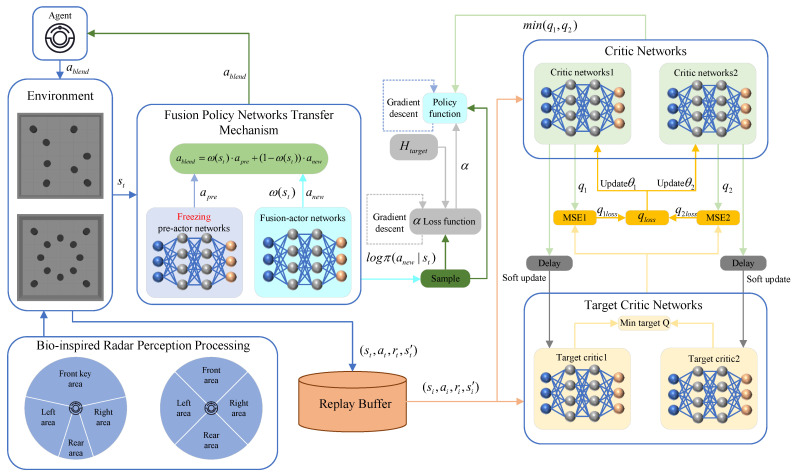
LT-SAC algorithm framework.

In nature, organisms typically integrate long-term experience with current sensory information to form highly adaptable and flexible behavioral strategies, enabling them to cope with environmental uncertainty and dynamics. Inspired by this, we propose a brain-inspired policy fusion reinforcement learning algorithm for robots (LT-SAC) within the Soft Actor–Critic (SAC) framework. This work introduces three key innovations: a brain-inspired policy fusion transfer mechanism that enables weighted integration of pretrained and current policies; a bio-inspired radar feature optimization method that enhances environmental perception and improves state representation quality; and a reward design based on ineffective behavior recognition, which effectively avoids ineffective exploration and local stagnation during training. All these methods are embedded within the SAC framework, as illustrated in the overall framework diagram in Figure 1. The Environment module represents the operating environment of the robot. The Bio-inspired Radar Perception Processing module denotes the regional segmentation of LiDAR data across different scenarios. The Fusion Policy Networks Transfer Mechanism module illustrates the process of generating fused actions. Replay Buffer serves as an experience replay pool for storing various data. The Critic Networks and Target Critic Networks are used to compute losses and prepare for parameter updates. The Policy function is employed to update the Fusion-actor networks. The α Loss function is responsible for adaptively adjusting the entropy parameter.

### 3.1. Transfer Learning Based on Fusion Policy Networks

#### 3.1.1. Core Principle of the SAC Algorithm

SAC is an off-policy reinforcement learning algorithm based on the maximum entropy framework, which balances policy performance and exploration capability. It demonstrates excellent learning stability and sample efficiency. Its objective function introduces an entropy regularization term into the conventional cumulative reward formulation, encouraging the policy to maintain sufficient randomness. The objective is expressed as follows:

(1) $$J\left(\pi \right)=\sum_{t=0}^{T-1}E(s_{t},a_{t})∼\rho \pi \left[r\left(s_{t},a_{t}\right)+\alpha H\left(\pi \left(\cdot |s_{t}\right)\right)\right]$$

Here, α is the temperature coefficient, which balances the importance between the reward and the entropy term. $H(\pi \left(\cdot |s_{t}\right))$ denotes the entropy of the policy at state $s_{t}$, defined as follows:

(2) $$H\left(\pi \left(\cdot |s_{t}\right)\right)=E_{a_{t}∼\pi }\left[-\log\pi \left(a_{t}|s_{t}\right)\right]$$

#### 3.1.2. Design of the Fusion Policy Networks

In the process of model transfer, traditional transfer learning methods heavily rely on the similarity between the source and target scenarios. When the complexity of the target environment is significantly higher than that of the source, the pretrained policy often fails to effectively guide the robot to complete path planning tasks in the new setting, resulting in negative transfer. To address this issue, this paper proposes a policy fusion networks-based transfer learning method (FPTM), which treats the pretrained policy as a representation of prior experience, while the current policy generates new actions corresponding to reactive behaviors based on real-time perception. By dynamically adjusting the fusion weights between these two components, the source policy can flexibly adapt to changes in the target environment, thereby improving the stability and generalization ability of policy transfer.

**Figure 2:**
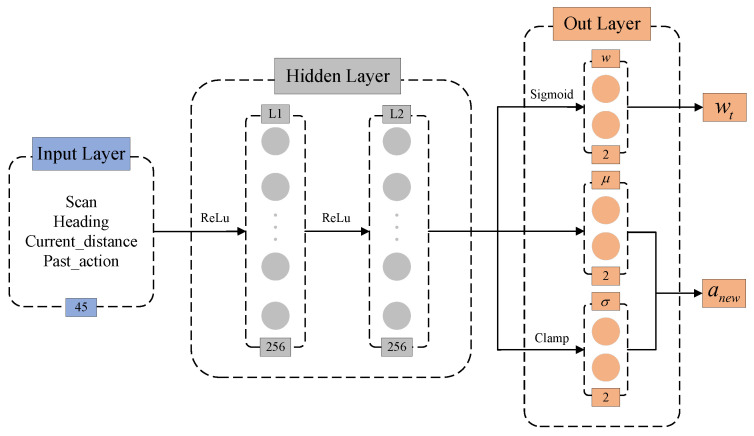
Fusion policy networks.

The fusion policy networks $\pi_{fusion}$ take the current state as input and outputs action-wise weights $w(s_{t})$ through a nonlinear mapping, which are used to combine the actions generated $a_{pre}$ by the pretrained policy and those generated by the current policy $a_{new}$, resulting in the final execution action $a_{blend}$. The networks adopts a two-layer fully connected neural networks (MLP), with ReLU activation functions in the hidden layers. The output layer employs a combination of Sigmoid and Clamp operations to constrain the output range, ensuring numerical stability and controllability. This mechanism retains prior knowledge while dynamically modulating exploratory behavior through learnable weights, thereby enhancing the flexibility and adaptability of policy transfer. The overall structure is illustrated in Figure 2.

#### 3.1.3. Fusion Policy Networks Transfer Mechanism

To enable efficient policy transfer, this section introduces the transfer mechanism of the fusion policy networks. First, the policy networks within the SAC framework processes the current state to extract an action $a_{pre}$ that represents the knowledge of the pretrained policy. Then, the fusion policy networks generate a new action $a_{new}$ and a corresponding weight factor $w(s_{t})$ based on the current environmental state. Finally, the pretrained action and the newly generated action are combined through a weighted fusion to produce the final execution action. The fused action $a_{blend}$ is then used to interact with the environment and serves as the basis for policy optimization, allowing the learning process to dynamically balance the influence between the prior and current policies. The process is described in Equation (3).

(3) $$a_{blend}=\omega \left(s_{t}\right)\cdot a_{pre}+\left(1-w(s_{t})\right)\cdot a_{new}$$

The training and update process of the fusion policy networks $\pi_{fuse}$ follows the core principles of the SAC algorithm, minimizing both the policy loss and Q-value loss. Through the automatic entropy tuning mechanism, it ensures that action fusion is achieved while maintaining sufficient exploration and stable convergence of the policy.

#### 3.1.4. Training and Update Process of the Algorithm

During training, after each iteration, a batch of interaction samples $\left(s_{i},a_{i},r_{i},s_{i}^{\prime }\right)$ is sampled from the replay buffer. At the next state $s_{t}^{\prime }$, a new action $a_{new}^{\prime }$ is generated by the fusion policy networks, which is then combined with the pretrained policy action $a_{pre}^{\prime }$ through weighted fusion to obtain the fused action $a_{blend}^{\prime }$.

To estimate the long-term return of state–action pairs, the LT-SAC algorithm adopts a double Q-networks structure and minimizes the mean squared error between the predicted and target Q-values. Since the objective of fusion policy training is to indirectly optimize the actions generated by the new policy—while the pretrained model remains frozen during the transfer process—the optimization of the action probability of $a_{new}$ effectively guides the fusion policy toward higher-value fused actions. This implicitly optimizes the weight function $w(s_{t})$, thereby enhancing the adaptability and generalization capability of the policy. The target Q-value is computed using the target networks $Q_{1}^{\prime }$ and $Q_{2}^{\prime }$, as follows:

(4) $${\hat{Q}}_{t}=r_{t}+\gamma \left(1-d_{t}\right)\left[\min\left(Q_{1}^{\prime }\left(s_{t}^{\prime },a_{new}^{\prime }\right),Q_{2}^{\prime }\left(s_{t}^{\prime },a_{new}^{\prime }\right)\right)-\alpha \log\pi \left(a_{new}^{\prime }|s_{t}^{\prime }\right)\right]$$

The update objective of the Q-networks is to minimize the following loss function, which is used to optimize the Q-networks parameters via gradient backpropagation:

(5) $$L_{critic}=E_{(s_{t},a_{t})∼D}\left[{\left(Q_{1}\left(s_{t},a_{t}\right)-{\hat{Q}}_{t}\right)}^{2}+{\left(Q_{2}\left(s_{t},a_{t}\right)-{\hat{Q}}_{t}\right)}^{2}\right]$$

The objective of policy optimization is to maximize the state value of the fused action $a_{new}$ while encouraging diversity in the outputs of the new policy. Therefore, the policy loss is defined as follows:

(6) $$L_{\pi }=E_{s_{t}∼D}\left[\alpha \log\pi \left(a_{new}|s_{t}\right)-\min\left(Q_{1}\left(s_{t},a_{new}\right),Q_{2}\left(s_{t},a_{new}\right)\right)\right]$$

To achieve an adaptive balance between exploration and exploitation, SAC introduces an automatic temperature tuning mechanism [38], which dynamically adjusts the temperature parameter α by minimizing the following loss function:

(7) $$L_{\alpha }=E_{a_{new}∼\pi }\left[-\alpha \left(\log\pi \left(a_{new}|s_{t}\right)+\bar{H}\right)\right]$$

The target entropy $\bar{H}$ in Equation (7) is a predefined scalar that determines the desired level of randomness in the policy’s action distribution. It acts as a regularization term, encouraging sufficient exploration during training. The definition of the target entropy is as follows:

(8) $$\bar{H}=-dim\left(A\right)$$

where dim(A) is the dimensionality of the action space. This formulation encourages maximum entropy for continuous actions.

### 3.2. Bio-Inspired Radar Perception Processing

#### 3.2.1. State Space

The fusion policy networks rely on accurate environmental representations to make informed decisions. To this end, we incorporate a LiDAR feature processing module that extracts compact and informative state features from raw sensor data.

In nature, organisms typically rely on multi-level perception and selective attention to critical regions to achieve efficient judgment and dynamic response in complex environments. Inspired by this, we propose a bio-inspired perception-driven LiDAR information optimization method (BIOM) that partitions the robot’s LiDAR field of view into regions and extracts multidimensional statistical features. This approach replaces the traditional single-point processing based solely on minimum distance, enhancing the robustness of environmental perception across scenarios of varying complexity and improving the state representation capability of the policy networks.

We first introduce the state space of the robot, which is composed of the following components:

(9) $$S_{t}=\left(scan,heading,current\_distance,past\_action\right)$$

Here, scan represents the environmental scan data obtained from the 2D LiDAR, which reflects the distance between the robot and surrounding obstacles. heading denotes the angular difference between the robot’s current orientation and the direction from the robot to the target point. The specific calculation is shown in Equation (10).

(10) $$\left\{\begin{array}{c} \theta_{goal}=atan2\left(y_{goal}-y_{robot},\;x_{goal}-x_{robot}\right)\\ heading=\theta_{goal}-\theta_{robot} \end{array}\right.$$

Here, $x_{robot}$ and $y_{robot}$ denote the current coordinates of the robot, while $x_{goal}$ and $y_{goal}$ represent the coordinates of the target point. $\theta_{robot}$ is the robot’s heading angle, obtained from quaternion conversion. Due to the periodic nature of angles, when calculating the angular difference between the robot’s orientation and the target direction, the error is normalized to the range [−π,π) to ensure continuity and physical meaning, thereby avoiding directional ambiguity. current_distance denotes the Euclidean distance between the robot’s current position and the target point. past_action represents the robot’s previous action, including angular velocity *w* and linear velocity *v*.

#### 3.2.2. LiDAR Region Segmentation Method

**Figure 3:**
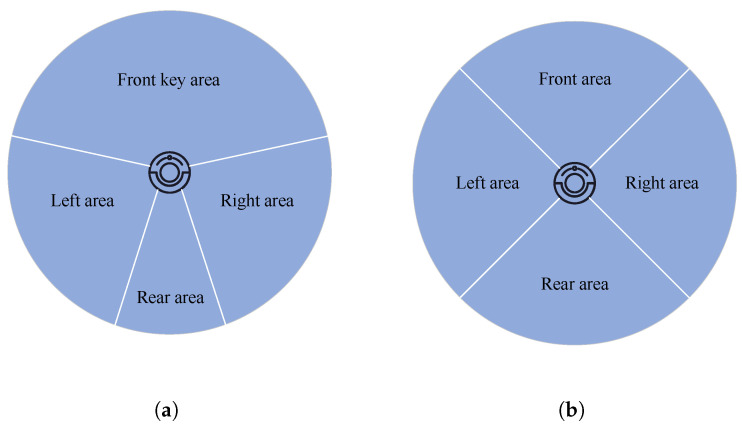
LiDAR region division diagram. (**a**) In static scenarios, the primary detection area is located in front of the robot. (**b**) In dynamic scenarios, obstacles may approach from any direction, so feature extraction must be performed in all directions.

This section presents optimizations for the LiDAR scan data in the state space. First, the laser data is sparsely sampled to reduce the dimensionality of the LiDAR input. Then, the field of view is divided into regions, as illustrated in Figure 3. Different importance levels are assigned to these regions depending on the scenario, extracting statistical features from the LiDAR data to compress the input while preserving the semantic information of obstacle distribution.

#### 3.2.3. Regional Feature Optimization Method

As mentioned earlier, the scan data from the LiDAR sensor on the TurtleBot3 mobile robot platform is included in the laser scan information, as shown in the following equation:

(11) $$scan=\left\{r_{1},r_{2},...,r_{i}\right\}$$

Each element $r_{i}$ represents the range value of the *i*-th laser beam, indicating the distance between the robot and an obstacle. The robot uses $\min\left\{r_{i}\right\}$ to determine whether it is too close to an obstacle.

Four statistical features—minimum, mean, standard deviation, and median—are extracted within each key region to comprehensively characterize the spatial structure in that direction, thereby enhancing the representational capacity of the LiDAR information.

(12) $$\left\{\begin{array}{cc} dim & =\min\{r_{1},r_{2},\ldots ,r_{i}\}\\ \mu & =\frac{1}{n}\sum_{i=1}^{n}r_{i}\\ \sigma & =\sqrt{\frac{1}{n}\sum_{i=1}^{n}{\left(r_{i}-\mu \right)}^{2}}\\ Med & =\mathrm{Median}\left\{r_{1},r_{2},\ldots ,r_{i}\right\} \end{array}\right.$$

Here, dim represents the minimum value, reflecting the distance to the nearest obstacle and serving as a critical signal for imminent collision. μ denotes the mean value, which evaluates overall passability; a higher mean indicates a relatively clear path. σ is the standard deviation, measuring environmental complexity and the uniformity of obstacle distribution; larger values indicate dense and unevenly distributed obstacles. Med stands for the median, which possesses strong noise robustness and provides a stable distance reference, thereby enhancing the reliability of obstacle avoidance strategies in complex scenarios.

### 3.3. Reward Function Based on Invalid Behavior Recognition Mechanism

#### 3.3.1. Reward Function

To fully utilize the enhanced state representation, we next design a reward function that encourages effective decision-making and penalizes inefficient behavior during training. The reward function is a critical component in robot navigation tasks, as it determines whether the robot can learn an effective policy. While most dense reward functions significantly aid exploration and task execution, they may cause the robot’s attention to become dispersed. For example, the robot may continuously receive rewards for facing and approaching the target point, which can lead to stagnation behaviors characterized by small action magnitudes and lack of movement. To address this issue, we propose a reward function based on an invalid behavior recognition mechanism (IBRM). The reward function is defined as shown in Equation (13).

(13) $$Reward=\left\{\begin{array}{cc} r_{goal}, & \mathrm{if}\;\mathrm{goal}\\ R_{d}+0.1\cdot R_{a}, & \mathrm{during}\;\mathrm{task}\;\mathrm{execution}\\ r_{collision}, & \mathrm{if}\;\mathrm{collision}\\ r_{stuck}, & \mathrm{if}\;\mathrm{the}\;\mathrm{action}\;\mathrm{is}\;\mathrm{invalid} \end{array}\right.$$

A positive reward $r_{goal}$ is given when the robot reaches the target point, and a negative reward $r_{collision}$ is assigned upon collision. To encourage the robot to progressively explore the environment and approach the target step by step, the position reached in the previous attempt is recorded and compared with the position reached in the current attempt. If the current position is closer to the target than the previous one, a positive reward is given; otherwise, a negative reward is assigned.

The definition of the distance influence factor $d_{rate}$ is as follows:

(14) $$d\_rate=d_{p}-d_{c}$$

$d_{p}$ denotes the position reached by the robot in the previous task execution, and $d_{c}$ denotes the position reached in the current task execution. The distance reward $R_{d}$ is defined as follows:

(15) $$R_{d}=\left\{\begin{array}{cc} d\_rate, & \mathrm{if}\;d\_rate>0\\ -0.5, & \mathrm{if}\;d\_rate\leq 0 \end{array}\right.$$

To further help the robot overcome the issue of sparse rewards in the early stages, an angular reward $R_{a}$ is provided based on the heading angle in the state space.

(16) $$R_{a}=\pi -\left|heading\right|$$

The variable heading represents the angular error between the robot’s current orientation and the target direction. It can be either positive or negative, depending on their relative directions. The absolute value is used to eliminate the effect of the sign.

#### 3.3.2. Invalid Behavior Recognition Mechanism

This section defines spatial and action constraints to detect stagnation during training. Spatially, the robot’s current and previous positions are $p_{t}=\left(x_{t},y_{t}\right)$ and $p_{t-1}=\left(x_{t-1},y_{t-1}\right)$, with the Manhattan distance $\Delta p_{t}$ used to avoid mistaking minor jitters for movement. If $\Delta p_{t}<\varepsilon_{p}$, the robot is considered stationary. In the action dimension, the policy outputs action $a_{t}=\left(v_{t},w_{t}\right)$ representing linear and angular velocities, with magnitude $\Delta a_{t}$. When $\Delta a_{t}<\varepsilon_{a}$, the policy fails to produce effective movement. If both spatial and action thresholds are met, the behavior is marked invalid, triggering stagnation detection.

(17) $$\left\{\begin{array}{cc} \Delta a_{t} & =\left|v_{t}\right|+\left|\omega_{t}\right|\\ \Delta p_{t} & =\left|x_{t}-x_{t-1}\right|+\left|y_{t}-y_{t-1}\right| \end{array}\right.$$

To avoid overreacting to occasional stationary behaviors, a counter $\Delta_{t}$ is introduced to record the number of consecutive steps that satisfy the above two stagnation conditions. If these two conditions hold continuously for *N* steps, the stagnation mechanism is triggered, an additional negative reward $r_{stuck}$ is given, and the current training episode is terminated early.

(18) $$\Delta_{t}=\left\{\begin{array}{cc} s_{t-1}+1, & \mathrm{if}\;\Delta p_{t}<\varepsilon_{p}\;\mathrm{and}\;\delta a_{t}<\varepsilon_{a}\\ 0, & \mathrm{otherwoise} \end{array}\right.$$

To present the overall method framework more clearly, the algorithm pseudocode is shown in Algorithm 1:
**Algorithm 1:** Pseudo-Code of LT-SAC Algorithm
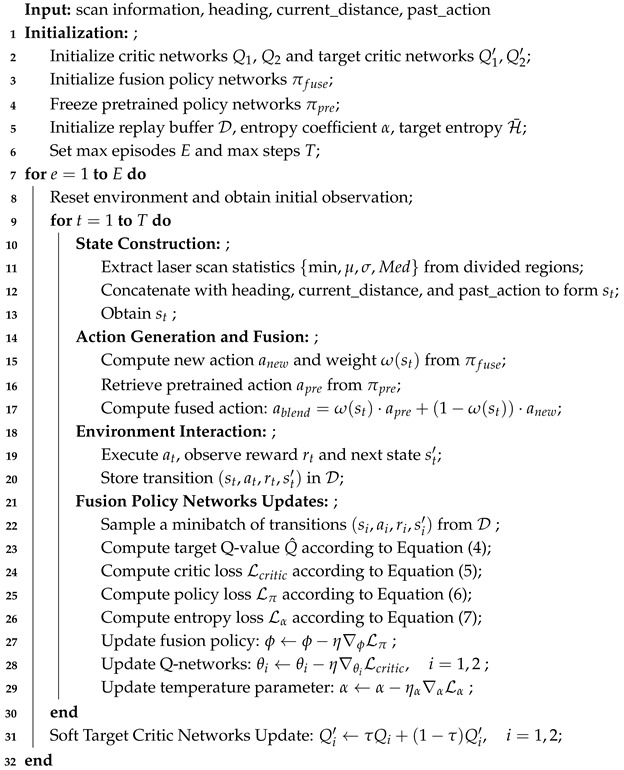


## 4. Experimental Validation

### 4.1. Experimental Setting

This section first conducts ablation studies on key modules, including the fusion policy networks and lidar information optimization, to validate their effectiveness. Then, the LT-SAC algorithm is evaluated through transfer experiments in environments of varying difficulty to assess its generalization. Experiments are based on the ROS framework and use the Gazebo simulator, developed by the Open Source Robotics Foundation (OSRF),a powerful 3D physics engine supporting realistic dynamics, sensor modeling, and robot interaction [39].

All experiments were performed on Ubuntu 20.04 with ROS Noetic using the TurtleBot3 platform equipped with a lidar sensor (3.5 m range). Each experiment was repeated multiple times with independent runs, and average metrics were recorded every 500 episodes for analysis. Transfer learning experiments used pretrained models, reducing total training episodes due to faster convergence. Detailed parameters are listed in Table 1.

#### Simulation Scenarios

**Figure 4:**
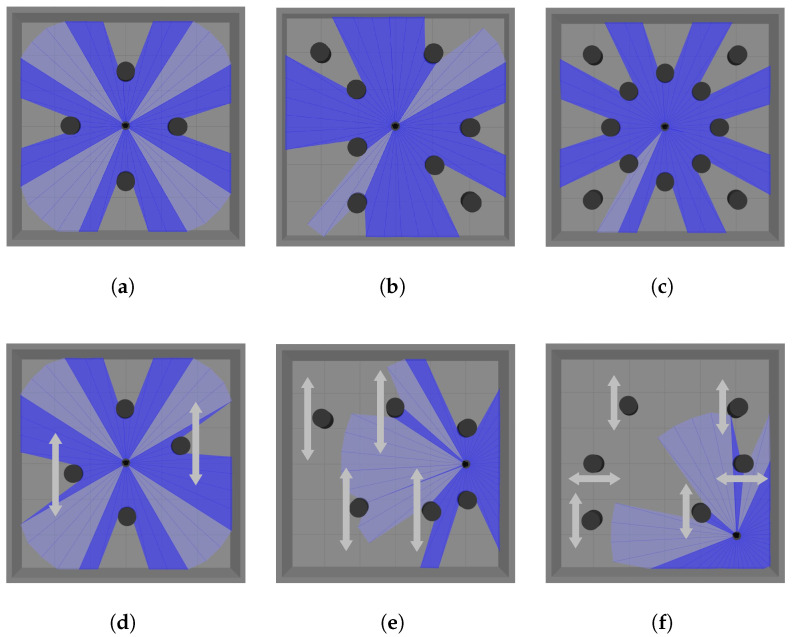
Different simulation scenarios. (**a**) The scenario with four static obstacles. (**b**) The scenario with eight irregularly distributed static obstacles. (**c**) The more complex scenario with an increased number of static obstacles, where the robot must navigate through narrower gaps. (**d**) The scenario with two static obstacles and two dynamic obstacles, where the arrows indicate the back-and-forth movement direction of the dynamic obstacles. (**e**) The scenario with four dynamic obstacles and two static obstacles. (**f**) The scenario with six dynamic obstacles.

To validate the effectiveness of the proposed policy transfer method in robot navigation, this paper designs a series of simulation scenarios with progressively increasing difficulty, covering both static and dynamic obstacle tasks. The scenarios gradually increase in the number, distribution density, and dynamic properties of obstacles, forming a task sequence from simple to complex. Figure 4 shows representative constructed scenarios, each measuring 6 m × 6 m. The robot’s position in the figure indicates the starting point for each training episode, while the goal point is randomly updated. The light blue and dark blue areas represent the visualized results of the LiDAR scan range in Gazebo. Specifically, when the LiDAR does not detect any obstacles within its maximum scanning range, it is displayed as light blue; otherwise, it is shown as dark blue.

### 4.2. FPTM Experimental Validation

This section aims to evaluate the transferability and generalization capability of the fusion policy networks across different environments and to analyze how the characteristics of pretraining scenarios influence policy generalization. Two pretrained models were developed, each trained for 1500 episodes in the source scenarios (a) and (d), respectively. These pretrained models were then transferred to multiple target scenarios for policy transfer experiments. Specifically, the scenario (a) pretrained model was transferred to the (b) and (c) scenarios, while the scenario (d) pretrained model was transferred to the (e) and (f) scenarios.

**Figure 5:**
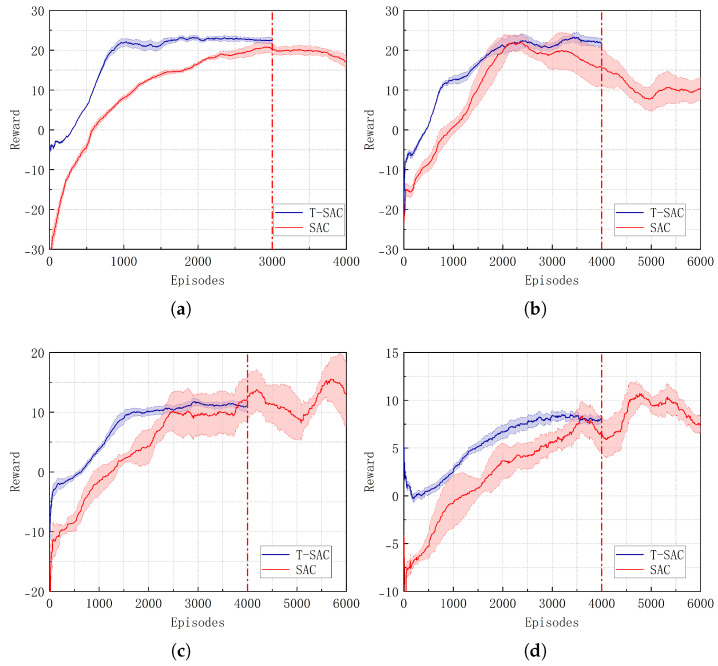
Performance of T-SAC and SAC in different scenarios. (**a**) In scenario (b). (**b**) In scenario (c). (**c**) In scenario (e). (**d**) In scenario (f).

Because the original SAC algorithm fails to learn an effective policy and achieve curve convergence in challenging scenarios, it is not possible to calculate the percentage of performance improvement or decline. As shown in Figure 5 and Table 2, in scenario (b), the SAC algorithm exhibits a relatively slow convergence rate during training and limited policy performance. In contrast, the T-SAC method enhanced with the FPTM improves the convergence rate by 46.67% and increases the overall return by 16.22%, demonstrating its effectiveness in policy transfer. Particularly in scenario (c), the SAC algorithm struggles to converge stably, whereas T-SAC shows stronger policy learning capabilities. In dynamic obstacle scenarios, as environmental complexity increases, the SAC algorithm exhibits noticeable instability and performance degradation, making it difficult to learn effective strategies. The T-SAC algorithm converges in 2500 and 3000 episodes in scenarios (e) and (f), respectively. Under the same training conditions, it demonstrates a more stable learning process and stronger adaptability to the environment, indicating that the fusion policy exhibits better generalization capability when handling tasks in dynamic scenarios.

### 4.3. BIOM Experimental Validation

**Figure 6:**
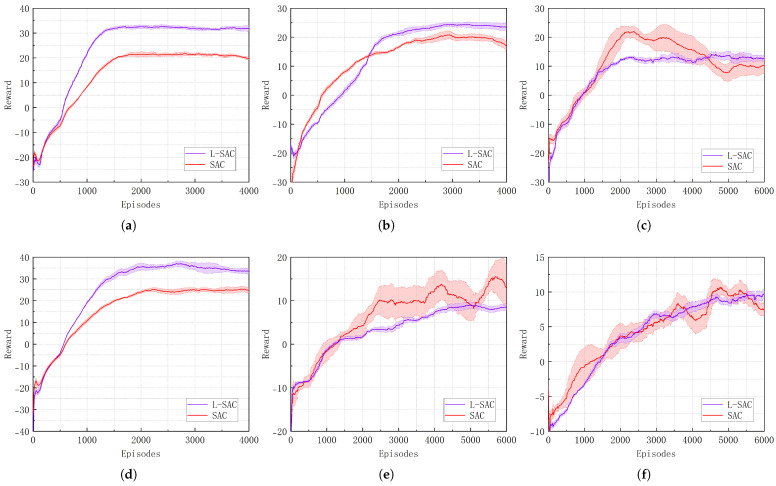
Performance of L-SAC and SAC in different scenarios. (**a**) In scenario (a). (**b**) In scenario (b). (**c**) In scenario (c). (**d**) In scenario (d). (**e**) In scenario (e). (**f**) In scenario (f).

This section aims to evaluate the effectiveness of the BIOM in reinforcement learning-based navigation tasks. To this end, comparative experiments are conducted in six simulated environments with varying levels of difficulty. Under identical training configurations, we compare the learning processes before and after the optimization, analyzing differences in terms of convergence speed, final average return, and overall training stability; this is illustrated in Figure 6 and Table 3.

As shown in Figure 6a in scenario (a), the L-SAC algorithm achieves convergence 11.76% earlier and outperforms the SAC algorithm in both stability and return, with a substantial improvement of 46.50% in terms of cumulative return. In the scenario with eight static obstacles (b), the L-SAC algorithm converges 200 episodes earlier than the SAC algorithm and achieves a 28.35% improvement in return. As the scenario complexity increases, the SAC policy exhibits greater fluctuations and struggles to converge. In contrast, in the more complex static obstacle scenario (c), the L-SAC algorithm converges in 2700 episodes with a return of 13.46, demonstrating stronger learning stability and the ability to maintain effective policy learning even in high-difficulty environments.

In dynamic obstacle scenarios, as shown in Figure 6d, scenario (d) features relatively few obstacles, allowing all algorithms to maintain stable policy learning. Compared to the original algorithm, L-SAC demonstrates faster convergence and better return performance, achieving a 9.09% improvement in convergence speed and a 38.95% increase in return. In scenarios (e) and (f), as the number of dynamic obstacles increases, the original SAC algorithm exhibits significant policy fluctuations and fails to converge. Although the convergence speed of the L-SAC algorithm decreases, reaching 4500 and 4700 episodes, respectively, it is still able to learn effective policies within a reasonable number of training episodes.

### 4.4. LT-SAC Experimental Validation

**Figure 7:**
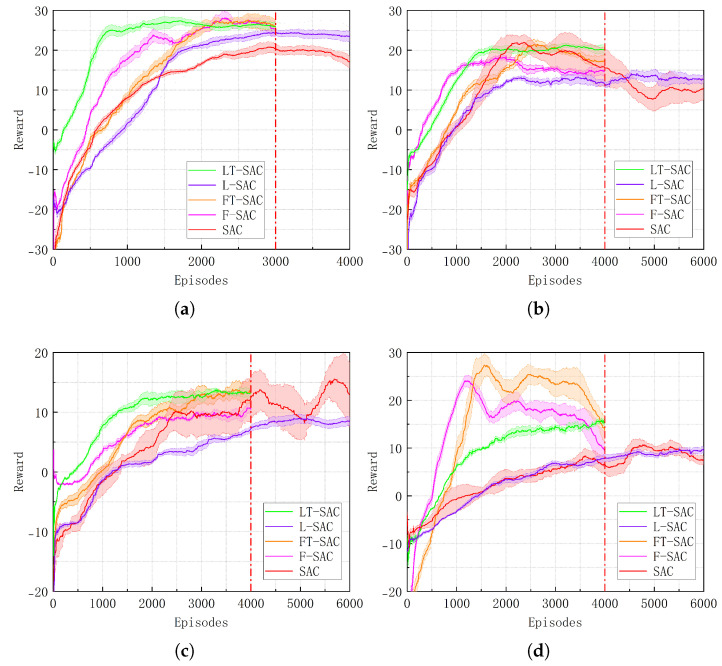
Performance of the five algorithms in different scenarios. (**a**) In scenario (b). (**b**) In scenario (c). (**c**) In scenario (e). (**d**) In scenario (f).

Finally, to further evaluate the transfer performance and decision-making efficiency of the proposed methods in complex environments, the three techniques are integrated into a unified framework to construct an optimized fusion policy model. To verify its effectiveness, the L-SAC algorithm is first trained for 1500 episodes in the source scenarios (a) and (d) to obtain pretrained policy models, which are then transferred to the four representative target scenarios (b), (c), (e), and (f) for evaluation. The results are compared against four baselines—T-SAC, SAC, FT-SAC, and F-SAC. A comprehensive analysis is conducted across multiple metrics, including convergence speed, cumulative reward, and policy stability, to assess the performance advantages of the fusion mechanism. During training, different LiDAR feature extraction mechanisms are applied according to each scenario. The experimental results are presented in Figure 7.

To further validate the proposed policy transfer method in reinforcement learning, a Unified Evaluation Metric (UEM) is introduced, which integrates two key aspects—task success rate and path length—into a unified framework for quantitative evaluation. Additionally, to assess the advantages of transfer learning, post-transfer return and convergence episodes are also incorporated into the metric. The mathematical definition of UEM is as follows:

(19) $$\mathrm{UEM}=\alpha S+\beta \cdot \frac{1}{1+L}+\gamma \cdot \frac{R}{1+|R|}+\delta \cdot \frac{1}{1+E_{s}}$$

where *S* denotes the success rate, *L* represents the average path length, *R* is the return value, and $E_{s}$ is the episode at which convergence begins. α, β, γ, and δ are weighting coefficients that satisfy α+β+γ+δ=1.

As shown in Figure 7, in static obstacle scenarios, all three transfer strategies exhibit good performance. Compared to the L-SAC method without transfer mechanisms, they achieve varying degrees of improvement in both convergence speed and cumulative return. However, in the dynamic scenario (e), the convergence speeds of FT-SAC and F-SAC decline significantly, indicating weaker adaptability. In the more complex scenario (f), due to frequent environmental changes and increased obstacle dynamics, both strategies experience noticeable fluctuations during training, reduced stability, and overall performance degradation, leading to negative transfer effects.

During the testing phase, the weighting coefficients in the comprehensive performance metric UEM were set as follows: the importance weight of the convergence episode indicator, δ, was increased to 0.4. The weights of the other three indicators were set equally as α=β=γ=0.2.

Considering that some algorithms may not converge within the training period in complex environments, the maximum number of training episodes is used as their convergence episode value. Since these algorithms do not converge and lack a valid final performance, the average return is adopted as the baseline for UEM calculation to ensure consistency and comparability of the metric.

The success rate test follows the same interaction mechanism as in training, conducting 100 independent trials in the simulation environment. In each trial, the goal position is randomly sampled, and the robot starts from the initial state. The task ends immediately upon either reaching the goal successfully or colliding. The number of successful completions is recorded to calculate the average success rate.

**Figure 8:**
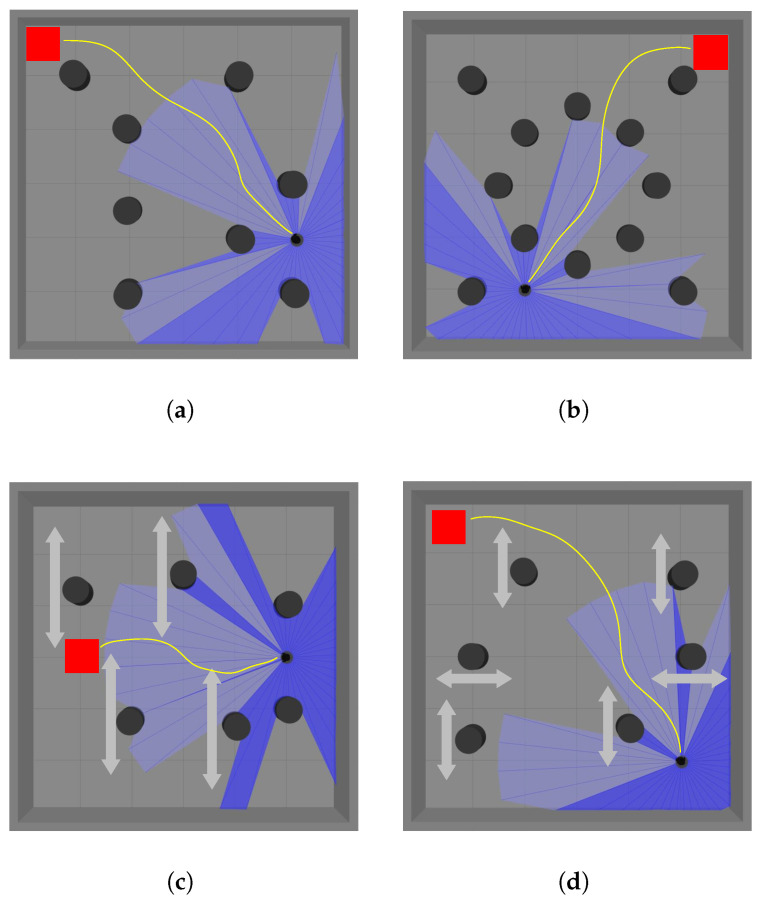
Path tests in different scenarios. The yellow curve represents an example trajectory for planning, and the red square indicates the location of the target point. (**a**) Testing in scenario (b). (**b**) Testing in scenario (c). (**c**) Testing in scenario (e). (**d**) Testing in scenario (f).

In the average path length test, the robot’s initial position and the goal position are fixed, as shown in Figure 8. This setup requires the robot to navigate through more obstacle areas during path planning. Under this configuration, 100 tests are conducted, recording the path length of each successful goal-reaching attempt and calculating the average path length over all valid trajectories.

**Figure 9:**
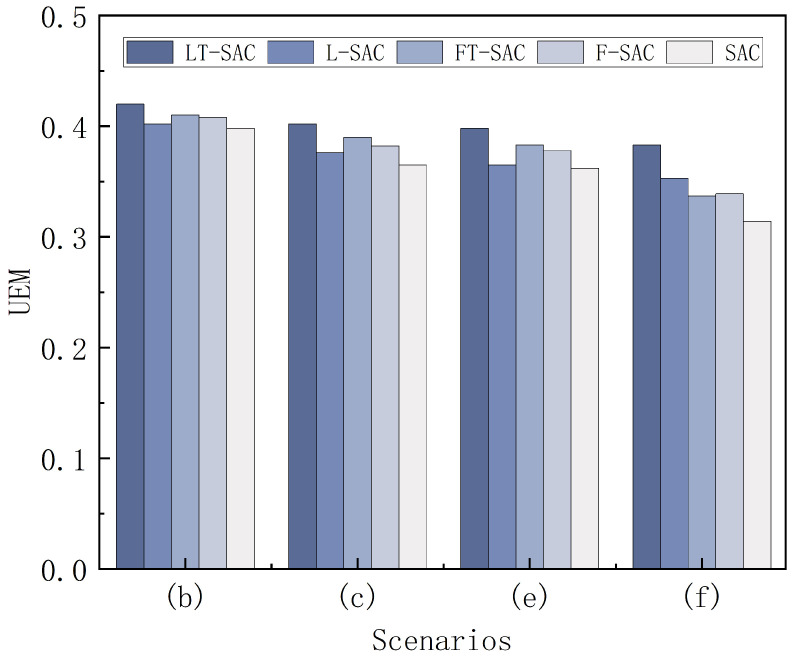
UEM indicators in different scenarios.

According to the testing methods described above, the test data statistics for different algorithms across various scenarios are presented in Table 4. The integrated performance metrics (UEM) of different algorithms in each scenario are illustrated in Figure 9.

As shown in the two figures above, LT-SAC achieves the highest UEM values across all scenarios, indicating its superior overall performance in terms of success rate, path optimization, policy stability, and convergence efficiency.

In the static obstacle scenarios (b) and (c), all four algorithms achieved good performance and successfully learned effective policies. In these two scenarios, LT-SAC obtained UEM scores of 0.420 and 0.402, respectively, with path planning success rates of 99% and 90%, outperforming the other algorithms. Additionally, LT-SAC showed a slight advantage in terms of path length. In the dynamic obstacle scenarios (e) and (f), the increased complexity of the environments led to a general performance decline across all algorithms compared to the static scenarios, with the most significant impact observed in convergence episodes. In scenario (f), both FT-SAC and F-SAC exhibited varying degrees of negative transfer, as the pretrained policies failed to effectively guide the robot in the target environment, resulting in lower success rates than L-SAC. In contrast, LT-SAC consistently maintained success rates above 80% and demonstrated stable convergence performance.

From a horizontal comparison, in complex static environments such as (c) and (f), LT-SAC achieved UEM scores of 0.402 and 0.383, significantly outperforming the other algorithms. This indicates that LT-SAC can effectively inherit the capabilities of the source policy and quickly adapt to target tasks, demonstrating strong transfer generalization even when the similarity between source and target environments is low. In comparison, although L-SAC is able to achieve convergence with the support of optimized statistical features from LiDAR information, its UEM values remain generally low across all scenarios. As for SAC, which serves as the baseline algorithm without any enhancements, its performance metrics are consistently the lowest in each scenario.

### 4.5. Actual Scenario Validation

Furthermore, the proposed method is validated in real-world scenarios. After completing policy pretraining in the simulation environment, the trained model is directly deployed on a real robotic platform for testing. The robotic platform used is the TARKBOT R10 (manufactured by Yantai TARK Electronics Technology Co., Ltd., Yantai, China), a two-wheel differential-drive mobile robot equipped with a Raspberry Pi 4B (manufactured by Raspberry Pi Foundation, Cambridge, UK) as the main control unit and a SLAMTEC A1 360° LiDAR (manufactured by Shanghai Slamtec Co., Ltd., Shanghai, China) for environmental perception.

#### 4.5.1. Actual Static Scenario

**Figure 10:**
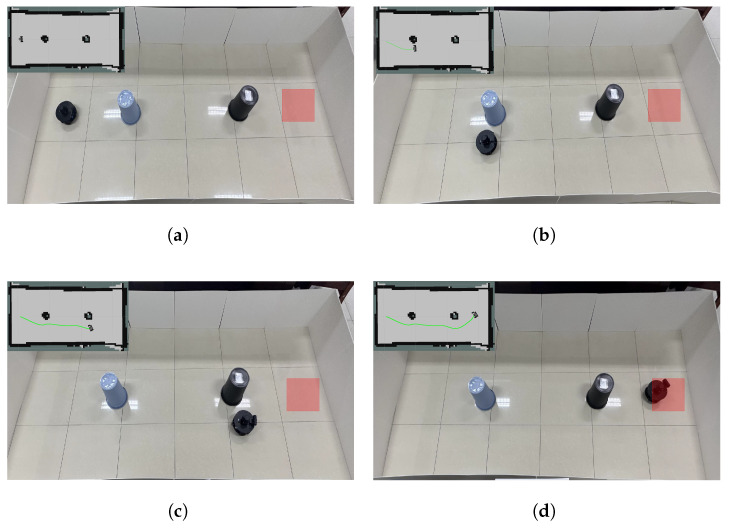
Static obstacle avoidance process in actual scenario. (**a**) Initial and target positions. (**b**) The robot avoids the first static obstacle. (**c**) The robot avoids the second static obstacle. (**d**) The robot reaches the target point.

The testing process is illustrated in Figure 10. The upper-left corner of the figure shows the robot’s movement within the scene, with the green curve representing the robot’s driving trajectory. The map was generated by the robot using its LiDAR sensor in a real experimental environment. It is important to note that the corresponding cost map is used solely for visualizing the robot’s trajectory in the real environment via RViz and does not participate in path planning or any form of navigation decision-making.

#### 4.5.2. Actual Dynamic Scenario

**Figure 11:**
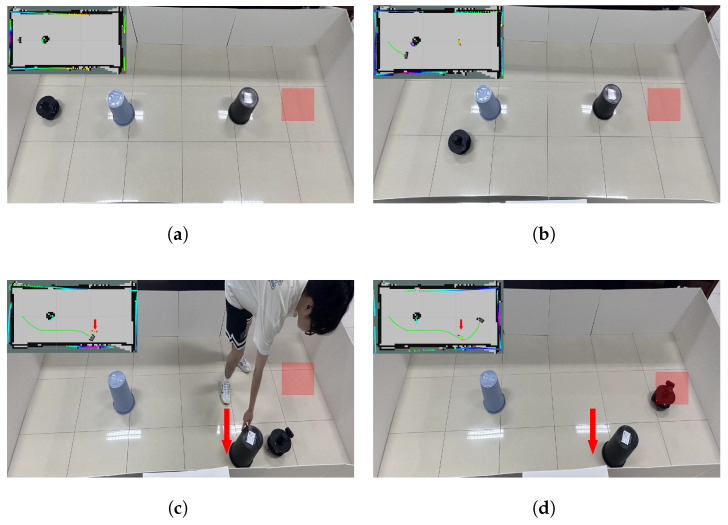
Mixed obstacle avoidance process in actual scenario. (**a**) Initial and target positions. (**b**) The robot avoids a static obstacle. (**c**) A dynamic obstacle approaches the robot; the robot turns to avoid it and passes through. (**d**) The robot reaches the target point.

**Figure 12:**
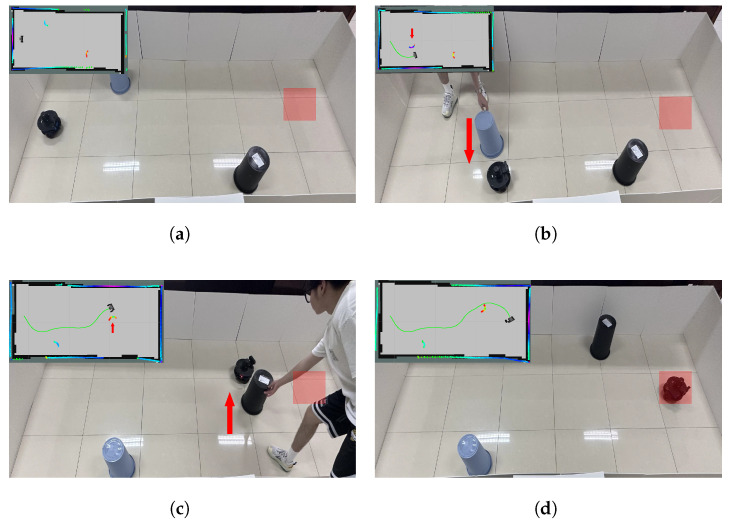
Dynamic obstacle avoidance process in actual scenario. (**a**) Initial and target positions. (**b**) The robot turns to avoid the first dynamic obstacle. (**c**) The robot turns to avoid the second dynamic obstacle. (**d**) The robot reaches the target point.

After completing real-world testing in the static obstacle scenario, the obstacle avoidance performance in dynamic environments was further evaluated. Based on the static obstacle scenario, a hybrid obstacle scenario was constructed by manually moving a second obstacle. To better visualize the behavior of dynamic obstacles, LiDAR feedback was used to render their motion, as shown in Figure 11. Building upon this, both obstacles were set to be movable to construct a fully dynamic obstacle scenario, as illustrated in Figure 12.

Through real-world robot testing, the effectiveness and robustness of the proposed method in the simulation-to-reality transfer process were further validated. Despite the inevitable challenges during deployment—such as variations in ground friction coefficients and sensor noise—which resulted in issues like relatively slower planning speeds and increased trajectory lengths, the overall system was still able to successfully perform obstacle avoidance and navigation tasks. This demonstrates the algorithm’s strong generalization capability and practical feasibility. The comprehensive testing results confirm that the fusion policy can maintain stable operation and effectively avoid obstacles even in real-world dynamic environments.

## 5. Conclusions

This paper addresses the transfer challenges faced by reinforcement learning in path planning tasks by proposing an action-level fusion transfer mechanism. This mechanism dynamically weights actions through fusion policy networks, thereby enhancing the generalization and adaptability of the policy in new environments. Additionally, a bio-inspired radar feature processing mechanism is introduced, applying different perception requirements for static and dynamic scenarios to improve the robot’s understanding of critical environmental information. A penalty design for ineffective behaviors is also implemented to improve the reasonableness of the policy’s actions in complex environments. Unlike existing hybrid SAC transfer learning frameworks that mainly rely on parameter- or feature-level transfer, our method performs fusion directly at the action output level, avoiding the difficulties traditional methods face in adapting to new environments. Meanwhile, the BIOM provides richer external sensory information for the robot in transfer scenarios. The IBRM better supervises the robot to learn effective policies.

Through experiments conducted in various static and dynamic simulation scenarios, the proposed fusion policy transfer method demonstrates excellent performance. Ablation studies show that both the FPTM and the BIOM contribute positively to the stability of policy learning. In scenario (b), the T-SAC algorithm improved the cumulative reward by 16.22% and enabled convergence in complex target environments. The L-SAC algorithm achieved convergence in all six scenarios, and in the dynamic obstacle scenario (d), it outperformed the baseline algorithm by 38.95% in terms of reward. Furthermore, the LT-SAC algorithm consistently outperformed SAC, FT-SAC, and F-SAC in terms of convergence speed, average reward, and success rate. In real-world robot experiments, the proposed method also exhibited strong robustness and practicality, confirming its feasibility in real environments.

Future research will further extend the application scope of the proposed transfer mechanism. In single-agent path planning, one promising direction is to explore dynamic obstacle avoidance, especially in scenarios involving sudden obstacle emergence, where the robot’s adaptive behavior holds significant research value. Additionally, the approach can be expanded to multi-agent cooperative control tasks, where managing inter-robot state information is crucial for enhancing obstacle avoidance capabilities in complex and dynamic environments. Moreover, narrowing the performance gap between simulation and real-world environments remains a valuable research direction, with the aim of improving the generalizability and reliability of learned policies in practical applications. 

## Figures and Tables

**Table 1:** Parameter setting.

Parameter Name	Symbol	Value
State Space Dimension	state_dim	45
Action Space Dimension	action_dim	2
Hidden Layer Dimension	hidden_dim	256
Replay Buffer Size	replay_buffer_size	50,000
Training Batch Size	batch_dim	256
Discount Factor	γ	0.99
Learning Rate	$l_{r}$	0.0001
Soft Update Coefficient	τ	0.005
Entropy	α	0.2
Velocity Coefficient	$\varepsilon_{a}$	0.1
Position Coefficient	$\varepsilon_{p}$	0.1
Goal Reward	$r_{goal}$	8
Collision Penalty	$r_{collision}$	−4
Penalty of Stagnation	$r_{stuck}$	−2
Stagnation Steps Threshold	*N*	4

**Table 2:** Performance comparison between T-SAC and SAC.

Scenario	Index	T-SAC	SAC	Improvement (%)
(b)	Episodes	1600	3000	+46.67
	Reward	23.43	20.16	+16.22
(c)	Episodes	2100	–	–
	Reward	22.57	–	–
(e)	Episodes	2500	–	–
	Reward	12.15	–	–
(f)	Episodes	3000	–	–
	Reward	8.47	–	–

**Table 3:** Performance comparison between L-SAC and SAC.

Scenario	Index	L-SAC	SAC	Improvement (%)
(a)	Episodes	1500	1700	+11.76
	Reward	32.86	22.43	+46.50
(b)	Episodes	2800	3000	+6.67
	Reward	24.54	19.12	+28.35
(c)	Episodes	2700	–	–
	Reward	13.46	–	–
(d)	Episodes	2000	2200	+9.09
	Reward	34.53	24.85	+38.95
(e)	Episodes	4500	–	–
	Reward	8.67	–	–
(f)	Episodes	4700	–	–
	Reward	9.94	–	–

**Table 4:** Test data of the algorithm in different scenarios.

Scenario	Algorithm	*S*	*L*	$E_{s}$	*R*	UEM
(b)	LT-SAC	0.99	5.66	1250	22.75	0.420
	L-SAC	0.91	5.72	3000	19.12	0.402
	FT-SAC	0.94	5.70	2000	22.46	0.410
	F-SAC	0.93	5.69	2200	22.94	0.408
	SAC	0.89	5.81	3000	20.16	0.398
(c)	LT-SAC	0.90	5.47	1500	20.52	0.402
	L-SAC	0.80	5.82	2700	13.46	0.376
	FT-SAC	0.85	5.74	2500	18.37	0.390
	F-SAC	0.82	5.62	2500	15.13	0.382
	SAC	0.74	5.93	6000	15.56	0.365
(e)	LT-SAC	0.88	4.72	2000	13.75	0.398
	L-SAC	0.76	4.94	4500	8.67	0.365
	FT-SAC	0.82	5.12	3000	13.96	0.383
	F-SAC	0.81	4.92	3000	10.13	0.378
	SAC	0.72	5.19	6000	12.74	0.362
(f)	LT-SAC	0.84	6.12	2000	14.53	0.383
	L-SAC	0.73	6.84	4700	9.94	0.353
	FT-SAC	0.61	7.36	4000	20.25	0.337
	F-SAC	0.64	7.47	4000	15.12	0.339
	SAC	0.56	7.54	6000	8.46	0.314

## Data Availability

The original contributions presented in this study are included in the article.

## Abbreviations

The following abbreviations are used in this manuscript:

FPTM	Fusion Policy Networks Transfer Learning Method
BIOM	Bio-inspired Perception-driven LiDAR Information Optimization Method
IBRM	Invalid Behavior Recognition Mechanism
LT-SAC	The SAC algorithm integrated with three core components
L-SAC	The SAC algorithm with only BIOM
T-SAC	The SAC algorithm with only FPTM
FT-SAC	The SAC algorithm based on fine-tuning transfer method
F-SAC	The SAC algorithm based on frozen-layer transfer method
